# Assessment of Biofilm Formation and Resistance to Imipenem and Ciprofloxacin among Clinical Isolates of *Acinetobacter baumannii* in Tehran

**DOI:** 10.5812/jjm.8606

**Published:** 2014-01-01

**Authors:** Ahya Abdi-Ali, Saghar Hendiani, Parisa Mohammadi, Sara Gharavi

**Affiliations:** 1 Department of Biology, Faculty of Science, Alzahra University, Tehran, IR Iran

**Keywords:** *Acinetobacter baumannii*, Biofilm, Susceptibility Test, Ciprofloxacin, Imipenem

## Abstract

**Background:**

Biofilms are communities of bacteria attached to the surfaces in an extracellular polymeric matrix which are associated with many chronic infections in humans. *Acinetobacter* spp. are emerging as a major cause of nosocomial infections and *Acinetobacter* *baumannii *is the predominant species associated with this kind of infections.

**Objectives:**

In the present study, the potential of biofilm formation of clinical isolates, *A*.* baumannii, *was assessed by using crystal violet method. Furthermore, susceptibility pattern of these strains to ciprofloxacin and imipenem was determined.

**Methods and Materials:**

Biofilm formation by 75 *A*.* baumannii* isolates was evaluated by using microtiter plate and tube methods and crystal violet staining. Tube method was carried out under static and shaking conditions. Then, the susceptibility of isolates to ciprofloxacin and imipenem was determined.

**Results:**

Results showed that in tube method under shaking, 22% of clinical isolates were strong biofilm producers while 23% of them were not able to form biofilms. In this experiment, 18% and 42% of isolates were considered as moderate and weak biofilm-forming strains, respectively. In microtiter plate tests, 18% of strains were strong-biofilm producers and 25% of them were notable biofilm producers. In this assessment, 10% and 47% were considered as moderate and weak biofilm-forming isolates, respectively. The susceptibility tests, using microdilution method, confirmed that 92% of these isolates were resistant and 6.6% were susceptible to ciprofloxacin, although these results for imipenem were 68% and 24%, respectively.

**Conclusions:**

It can be concluded that most of *A. baumannii* isolates can form biofilm in microtiter plate and tube. The results also verified that most of these isolates were resistant to ciprofloxacin and imipenem.

## 1. Background

Biofilms are highly structured communities of bacteria enclosed to the surfaces that are attached in an extracellular polymeric matrix, exhibiting a modified phenotype compared with corresponding planktonic cells, especially in gene transcription, as well interaction with each other. This structure is identified as a common cause of human infection (1-4). Bacterial biofilms have been found on the surface of different instruments such as intubation tubes, catheters and artificial heart valves in addition of water pipe lines and cleaning instruments (2). The surfaces are usual targets of complex microbial communities (5). 

*Acinetobacter *spp. are ubiquitous, non-fermentative Gram negative bacteria, able to colonize in patients in intensive care units. *Acinetobacter baumannii *is the main species related to outbreaks of nosocomial infections. This microorganism is an important opportunistic nosocomial pathogen causing epidemic pneumonia, urinary tract infections, septicemia and meningitis (6). Epidemic strains of *A. baumannii *are noted for both intrinsic resistance to antibiotics and their abilities to acquire genes, encoding resistance determinants. Main mechanisms of resistance to β-Lactams and aminoglycosides are through the production of β-lactamases and aminoglycoside-modifying enzymes. Moreover, diminished expression of outer membrane proteins, mutations in topoisomerase and up-regulation of efflux pumps play important roles in antibiotic resistance (7).

According to the epidemiologic studies, *Acinetobacter* biofilms play a role in infectious diseases such as cystic fibrosis, periodontitis, bloodstream infection and urinary tracts infection because of their ability to indwell medical devices (8). 

Carbapenems are the preferred treatment for severe *Acinetobacter* infections, and have reserved better potential effect than other antimicrobial agents. During the last few years, carbapenem-resistant *Acinetobacter* isolates have been reported all over the world (9). In 2001, the international network for the study and prevention of emerging antimicrobial resistance (INSPEAR) define the emergence of carbapenem resistance in *Acinetobacter* as a ‘global sentinel event’, warranting prompt epidemiological and microbiological interventions (8).

Over the last decades, a broad range of models have been explained for the *in vitro* study of biofilm formation and development (10). These can be grouped into biofilm biomass assays (based on the quantification of matrix and both living and dead cells), viability assays (based on the quantification of viable cells) and matrix quantification assays (based on the specific staining of matrix components).

## 2. Objectives

In the present study, the potential of biofilm formation by 75 clinical *A. baumannii *strains, isolated from burn wounds and urinary catheters was evaluated by using microtiter plate and tube methods and crystal violet staining, and the susceptibility of the isolates to ciprofloxacin and imipenem was determined.

## 3. Materials and Methods

### 3.1. Bacterial Strains and Cultural Conditions

A total of 75 *A. baumannii *isolates were collected from different clinical sources, including burn wounds (from burn hospital) and urinary catheters (from an army hospital) and evaluated. Frozen stocks were prepared in Skim milk (Merck, Germany) containing 15% glycerol (Merck, Germany) and were stored at -70 ° C. All isolates were transferred from the stock cultures into Tryptic soy agar (TSA) (Merck, Germany) and were aerobically incubated at 37°C for 24 hours. *Pseudomonas aeroginosa* PA01 was used as positive control for biofilm formation tests and *P*.* aeroginosa* ATCC 27853 served as quality control for susceptibility tests.

### 3.2. Biofilm Formation 

Cultures were inoculated in Tryptic soy broth (TSB) (Merck, Germany) and adjusted to 0.5 McFarland standards. Each three wells of a non-adherence, sterile 96-well flat-bottomed were filled with 200 μL of bacterial suspension. Negative controls contained only TSB. Then, plates were covered and aerobically incubated for 24 hours at 37°C. Afterward, the content of each well was aspirated, rinsed five times with 250 μL of sterile physiological saline, emptied and left to dry. Then, the plates were stained for 5 minutes with 0.2 mL of 2% crystal violet (Merck, Germany). The excess of the stain was rinsed off by insertion the plate under running tap water. Later the plates were air dried; the dye bound to the adherent cells was resolubilized with 160 μL of 33% (v/v) glacial acetic acid. By using an ELISA reader, the OD of each well measured at 650 nm (10, 11).

Biofilm was also formed in test tubes. For this reason, 0.1 mL of bacterial culture obtained as above mentioned, was transferred to glass test tubes containing 10 mL TSB. The cultures, under two different conditions: agitation at 200 rpm. and stationary were incubated at 37 °C for 72 hours, following which the medium was removed and tubes were washed with distilled water, air dried and biofilm formation were assayed by crystal violet (7). All tests were carried out in triplicates. 

### 3.3. Statistical Analysis

SPSS 16 and one way ANOVA were used to calculate the differences between microtiter plate and tube methods under shaking and static conditions. Experiment was performed in triplicate. P values of ≤ 0.05 were considered as significant.

### 3.4. Susceptibility Testing

All tests were carried out in duplicates. Minimum inhibitory concentrations (MICs) were carried out according to the clinical laboratory standards institute (CLSI) guidelines by using microtiter plate method. To this reason, colonies from TSA were suspended in Muller Hinton broth (Merck, Germany) and adjusted to 0.5 McFarland and 1:100 diluted in Muller Hinton broth to reach to a final concentration of 1×10⁶ CFU/mL. 

Dilutions of ciprofloxacin and imipenem (Exir, Iran) were made in distilled water and phosphate buffer 0.01 M, respectively. The antibiotics were prepared at different concentrations ranged from 0.25 to 512 µg/mL. Each well filled with 100 µL of different dilution of the antibiotic and 100 µL of bacterial suspension. Microtiter plates were incubated at 37 ° C for 24 hours. MIC was determined as the minimum antibiotic concentration that inhibited the visible growth (12).

## 4. Results

The results determined the rate of adherence of the cells and ability to form biofilm of *Acinetobacter* isolates. Summarized results of the tube and microtiter-plate tests are presented in Table 1. Statistical analyses were carried out and the tube test and microtiter-plates showed significantly different results. For a comparative analysis, we used classification of adherence.

The adhesion of isolates is classified into four categories. Strains were classified as follows (10):

- OD ≤ ODc = Non-adherent
- ODc < OD ≤ 2ODc = Weakly adherent
- 3ODc < OD ≤ 4ODc = Moderately adherent
- 4OD < ODc = Strongly adherent
- ODc = OD of control

Results are shown in Table 1. All of isolates were placed in four different categories: strong, moderate, weak and nonbiofilm producers.

**Table 1.** Percent of *A*.* baumannii* Isolates Formed Biofilm in Test Tubes and Microtiter Plates

Test	Isolates, %			
	Weak Biofilm	Moderate Biofilm	Strong Biofilm	No Biofilm Producer
**Test tubes**				
Shaking condition	42	18	22	18
Static condition	40	16	21	23
**Microtiter plates**	41	10	18	25

**Figure 1.**
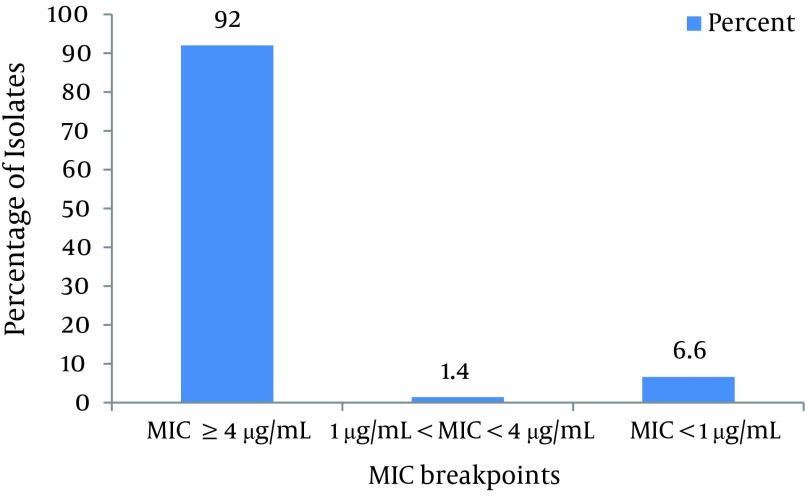
MIC of Ciprofloxacin

**Figure 2.**
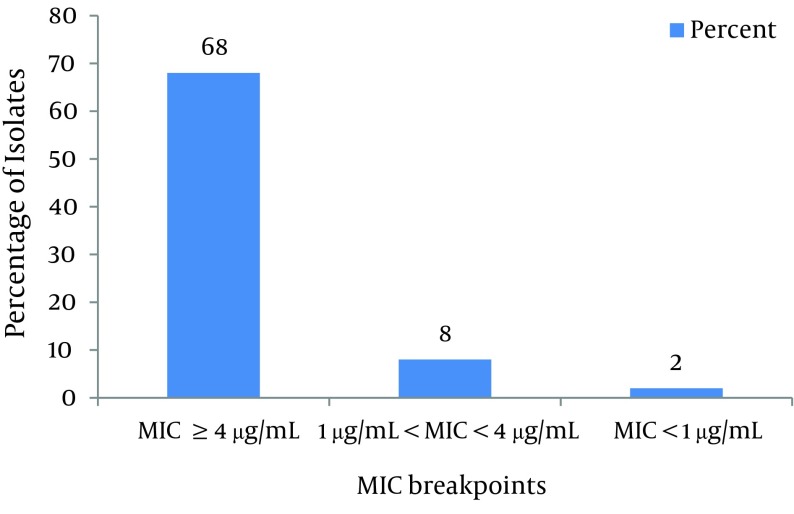
MIC of Imipenem

As shown in Figure 1 and according to CLSI protocol, the MIC breakpoint of ≥ 4µg/mL shows ciprofloxacin resistance and ≤ 1µg/mL susceptibility to this antibiotic. Consequently, 92% of isolates were resistant, 6.6% of isolates were susceptible and 1.4% of isolates were not susceptible or resistant to ciprofloxacin. As shown in Figure 2, based on CLSI tabletable 2B-2 MICs that are ≤ 4 g/mL are susceptible, and ≥ 16 g/mL are resistant. Therefore, 68% of isolates were resistant to imipenem, 24% were susceptible and 8% were not susceptible or resistant.

## 5. Discussion

Biofilms play a major role in microorganism colonization during infection, providing an opportunity for bacteria to develop drug resistance (12). According to the results of the tube tests, approximately all tested strains formed biofilm. The tube adherence assay is not complicated and simple although reading of the results may be complicated. Moreover, observers regularly have different interpretations about weak reactions (10). However, we noted that certain modifications of the process might improve the precision of interpretation of the results obtained by the tube test, e.g. after resolublizing, 200 µL of acetic acid was transferred to a well of a microtiter plate and read by ELISA plate reader, subsequently the approach was changing from a qualitative to a quantitative one.

The quantitative microtiter-plate method predicts clinical applications more reliable than the tube testing (7). In this study, significant disagreement between the tube test results and microtiter-plate was observed. Considerably more strains were classified as weak adherent by the quantitative microtiter-plate test. Factors that may influence the adherence of *Acinetobacter* are the hydrophobicity of the test tubes and the shaking which increases the chances of microorganisms interaction with the glass surface and uniform dispersion of the nutrients. 

Microtiter plate method is a very susceptible, precise, reproducible and affordable method for screening the biofilm formation and can function as a reliable quantitative method for determining biofilm formation. Biofilm formation by *A.* *baumannii* might increase the colonization and persistence of bacteria that may lead to higher rates of device related infections. In our microtiter-plate method, the addition of acetic acid permits to measure the attached bacteria both to the bottom and walls of the wells. Only 160 μL of 33% (v/v) glacial acetic acid was added per well, to evade interference with stained matter at the liquid–air interface, which was not considered to be indicative of biofilm formation in the tube tests (10).

Resistance patterns amongst nosocomial bacterial pathogens may generally be different from country to country and within a country over time. Because of these differences, a surveillance of nosocomial pathogens resistance is required for each country to show suitable selection for empiric therapy. In addition resistance monitoring could be predict an outbreak. Detection of resistance in a particular pattern may propose a presently occurring epidemic in the hospital (12).

Antibiotic resistance is the main cause of treatment failure of infected patients with all *Acinetobacter* species, particularly those with *A. baumannii* (13, 14). The first line therapy for *Acinetobacter* infections are amikacin, imipenem, ceftazidime, or a quinolone. Imipenem monotherapy have also been confirmed to be effective (15). However, many current studies have reported the increasing resistance to imipenem (16, 17). Most of the latest reports advocated the combination therapy in the present situation to avoid further resistance to imipenem, the antibiotic once considered as the drug of choice for *Acinetobacter* infections (15).

This high level of resistance to ciprofloxacin may be clarified by the fact that ciprofloxacin has been extensively used in hospitals. Chang et al. (8) reported the highest activity of quinolones against *A. baumannii* with 97.8% susceptibility. Imipenem has high affinity to the PBP2 of Gram-negative bacteria, and it has been reported to downregulte the expression of PBP2 which is associated to reduced susceptibility or resistance to carbapenems (18).

The ability of *A. baumannii* to construct or form biofilms could cause a high level of antibiotic resistance and survival properties (19). This possibility is supported by a very limited number of publications which offered that a clinical isolate of this bacterium is able to attach and form biofilms on glass surfaces (20, 21). *A.* *baumannii* isolates are capable to form biofilms might be selected under antibiotic pressure, or conversely, *A.* *baumannii* might acquire resistance to multiple drugs from the biofilm communities. In either case, it appears that the high colonization capacity of *A.* *baumannii*, combined with its resistance to multiple drugs, contributes to the organism survival and further dissemination in the hospital setting (22).

In conclusion, the results of study on the formation of biofilms in microtiter plates and test tubes under agitation and stationary growth conditions as well as statistical analysis indicated that the biofilms are formed most frequently under stationary conditions. Isolates of *Acinetobacter* from urinary catheters were more susceptible to the antibiotics than wound isolates, but isolates from burn wounds were stronger biofilm former than urinary catheters isolates. Multi-drug resistance to antibiotics in wound isolates is just because of the misuse of these antibiotics. Consequently, there is a direct relation between increased biofilm formation and antibiotic resistance. 
